# Fibromuscular Dysplasia as a Cause of Secondary Hypertension: A Case Report

**DOI:** 10.7759/cureus.99860

**Published:** 2025-12-22

**Authors:** Jorge Governa, Beatriz Marquês, Pedro Agostinho, Maria Gomes, Vasco Fonseca, Luzia Amaro Bismarck, Ana C Ribeiro, José Vale

**Affiliations:** 1 Internal Medicine, Unidade Local de Saúde do Oeste, Torres Vedras, PRT; 2 Internal Medicine, Centro Hospitalar do Oeste, E.P.E., Torres Vedras, PRT; 3 Neuroradiology, Unidade Local de Saúde de Santa Maria, Lisbon, PRT; 4 Neurology, Hospital Beatriz Ângelo, Lisbon, PRT; 5 Internal Medicine, Hospital CUF Torres Vedras, Torres Vedras, PRT

**Keywords:** fibromuscular dysplasia, ischemic stroke, pontine infarction, renal artery stenosis, secondary hypertension, vascular imaging, vertebral artery occlusion

## Abstract

Fibromuscular dysplasia (FMD) is characterized by the proliferation of connective tissue and smooth muscle fibers within the arterial wall, without inflammatory or atherosclerotic components, leading to stenosis, occlusion, or aneurysm formation and consequent impairment of perfusion in the affected organ. The renal, internal carotid, and vertebral arteries are most frequently involved. Secondary arterial hypertension and stroke are common clinical manifestations of FMD, as illustrated in the present case. A 39-year-old leucodermic male with a history of arterial hypertension, treated with amlodipine 10 mg and valsartan 160 mg, with poor compliance, presented to the emergency department with a one-week history of left-sided weakness and inability to walk. On examination, he was hypertensive (189/98 mmHg), with normal cardiac and pulmonary auscultation, left-sided hemiparesis predominantly affecting the leg, and a hemiparetic gait. Initial and 24-hour follow-up brain CT scans showed no evidence of acute ischemic or hemorrhagic lesions. ECG revealed sinus rhythm and voltage criteria for left ventricular hypertrophy. Echocardiography demonstrated left ventricular hypertrophy, left atrial dilation, and mild mitral regurgitation. Transcranial Doppler showed no right-to-left shunt. Brain MRI revealed an ischemic lesion in the right side of the pons, and magnetic resonance angiography demonstrated the absence of flow in the V4 segment of the left vertebral artery and reduced caliber of the left internal carotid artery. Laboratory tests showed worsening renal function after initiation of angiotensin-converting enzyme inhibitor therapy. Thrombophilia screening was negative. Due to suspected renal artery stenosis, CT angiography was performed, revealing saccular dilatations in both renal arteries with a “string-of-beads” appearance, suggestive of FMD. The patient was managed medically with blood pressure control. He began motor rehabilitation with partial recovery of neurological deficits and was discharged for follow-up in the outpatient clinic. In this patient, FMD was the underlying cause of the ischemic stroke that prompted hospitalization. The coexistence of difficult-to-control hypertension and target-organ damage in a young adult raised suspicion of secondary hypertension, and subsequent etiologic investigation led to the diagnosis of FMD.

## Introduction

Fibromuscular dysplasia (FMD) is an idiopathic, non-atherosclerotic, and non-inflammatory vascular disease characterized by abnormal cellular proliferation and disorganization of the arterial wall architecture in medium-sized arteries [1]. The condition most commonly affects the renal, extracranial carotid, and vertebral arteries, although virtually any arterial territory may be involved, resulting in stenosis, occlusion, aneurysm formation, or arterial dissection [2]. Clinically, FMD frequently presents with secondary (renovascular) hypertension and, when cerebrovascular arteries are affected, it may lead to transient ischemic attacks, ischemic stroke, or subarachnoid hemorrhage [2].

Although previously considered rare, recent evidence from large registries suggests that FMD remains underdiagnosed, particularly among young adults presenting with hypertension or neurological events [3]. The classic “string-of-beads” angiographic appearance reflects alternating segments of stenosis and arterial dilatation, and is most commonly associated with the medial form of the disease [1]. In young patients with difficult-to-control hypertension and cerebrovascular involvement, FMD should be considered in the differential diagnosis of secondary hypertension and stroke.

We present a case of a 39-year-old male in whom FMD, involving both the renal and cerebrovascular arteries, was the underlying cause of a pontine ischemic stroke. This case highlights the importance of etiologic investigation in young adults with target-organ damage and secondary hypertension, even in the absence of traditional atherosclerotic risk factors, particularly when surgical treatment may be warranted.

## Case presentation

A 39-year-old leucodermic male with a history of arterial hypertension, treated with amlodipine 10 mg and valsartan 160 mg, with poor compliance, presented to the emergency department with a one-week history of progressive weakness of the left hemibody associated with gait difficulty. He denied headache, visual changes, or sensory disturbances.

On admission, physical examination revealed that he was alert, oriented, afebrile, and hypertensive (189/98 mmHg). Cardiac and pulmonary auscultation were unremarkable. Neurological examination showed left hemiparesis, predominantly crural, with muscle strength graded as 4/5 in the left upper limb and 3/5 in the left lower limb, and a hemiparetic gait, without cranial nerve deficits or sensory abnormalities.

**Figure 1:**
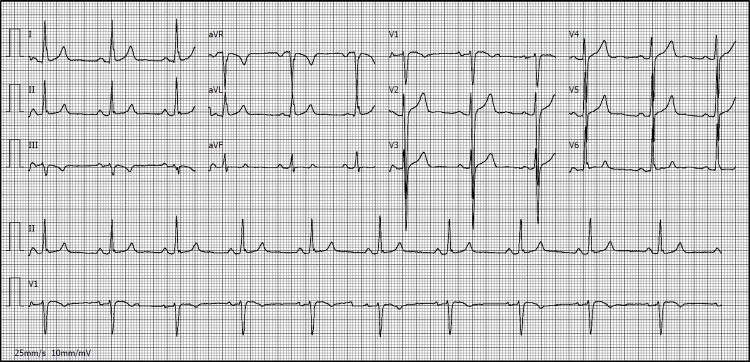
Electrocardiogram demonstrating sinus rhythm with left ventricular hypertrophy and lateral strain pattern. The 12-lead electrocardiogram showed sinus rhythm, voltage criteria for left ventricular hypertrophy (Sokolow-Lyon), and a lateral repolarization abnormality consistent with left ventricular strain (noted in leads I, aVL, V5–V6). No acute ischemic changes or arrhythmias were observed.

**Figure 2:**
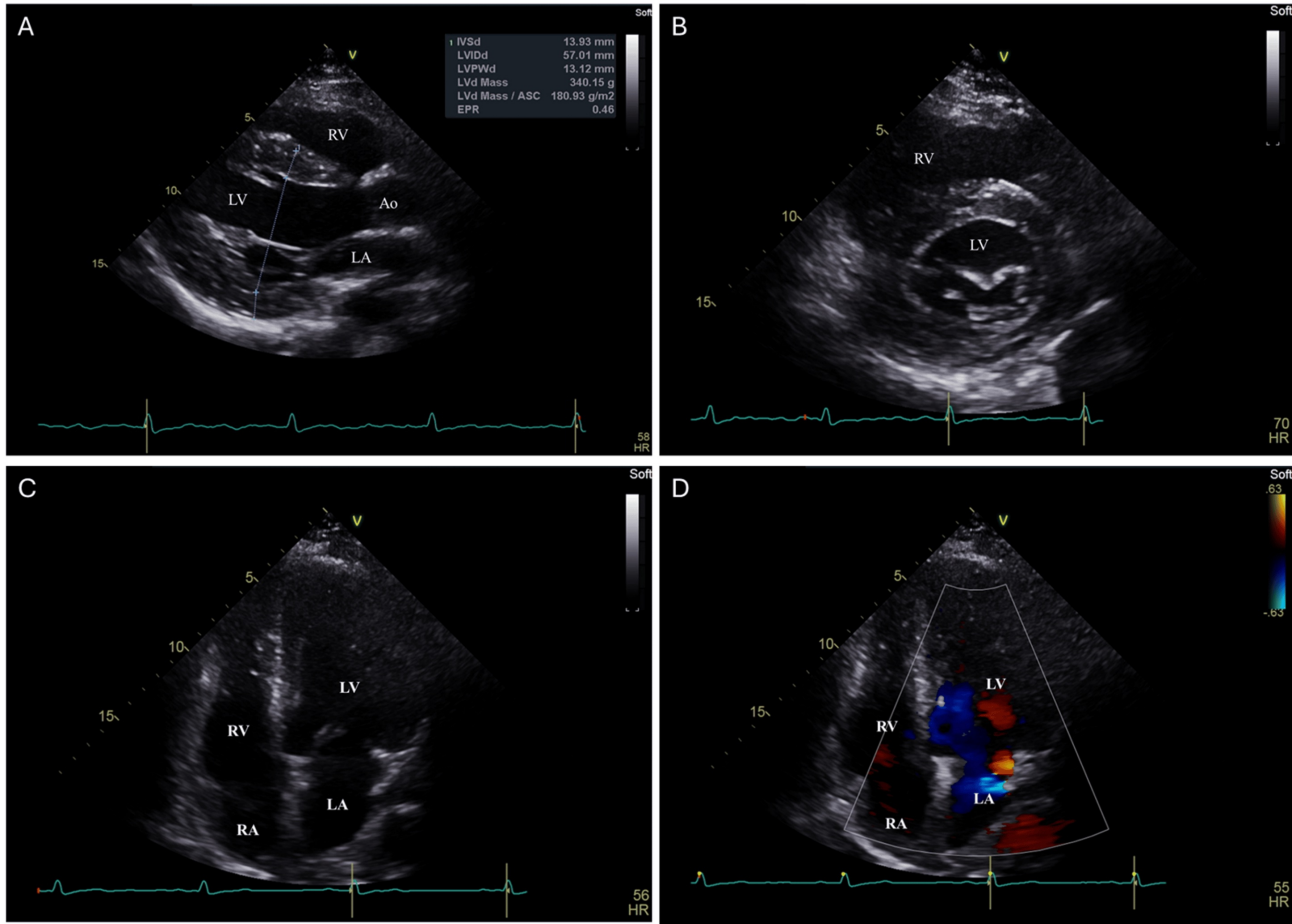
Echocardiographic evaluation demonstrating concentric left ventricular hypertrophy, mild left atrial dilation, and mild mitral regurgitation. A. The parasternal long-axis view showed increased thickness of the interventricular septum and posterior left ventricular wall (diastolic left ventricular posterior wall diameter: 13.12 mm), with a relatively small ventricular cavity, consistent with concentric left ventricular hypertrophy. B. Mid-ventricular parasternal short-axis view demonstrated uniform circumferential thickening of the left ventricular walls, compatible with concentric hypertrophy associated with long-standing hypertension. C. The apical four-chamber view revealed a mildly enlarged left atrium (left atrial volume index: 40 mL/m^2^) and increased left ventricular wall thickness. No significant structural abnormalities of the mitral valve were visible in this grayscale view. D. Color Doppler in the apical four-chamber view showed a small posteriorly directed mitral regurgitation jet, consistent with mild regurgitation, with otherwise normal appearance of the mitral leaflets. Ao: aorta; LA: left atrium; LV: left ventricle; RA: right atrium; RV: right ventricle.

Following the physical examination at admission, non-contrast cranial computed tomography (CT) showed no evidence of acute ischemic or hemorrhagic lesions, and a 24-hour follow-up CT was also normal. The electrocardiogram revealed sinus rhythm with voltage criteria for left ventricular hypertrophy (Figure 1). Transthoracic echocardiography confirmed concentric left ventricular hypertrophy, mild left atrial dilation, and mild mitral regurgitation (Figure 2). Transcranial Doppler ultrasound did not demonstrate a right-to-left shunt, and Doppler ultrasound of the cervical vessels was normal.

**Figure 3:**
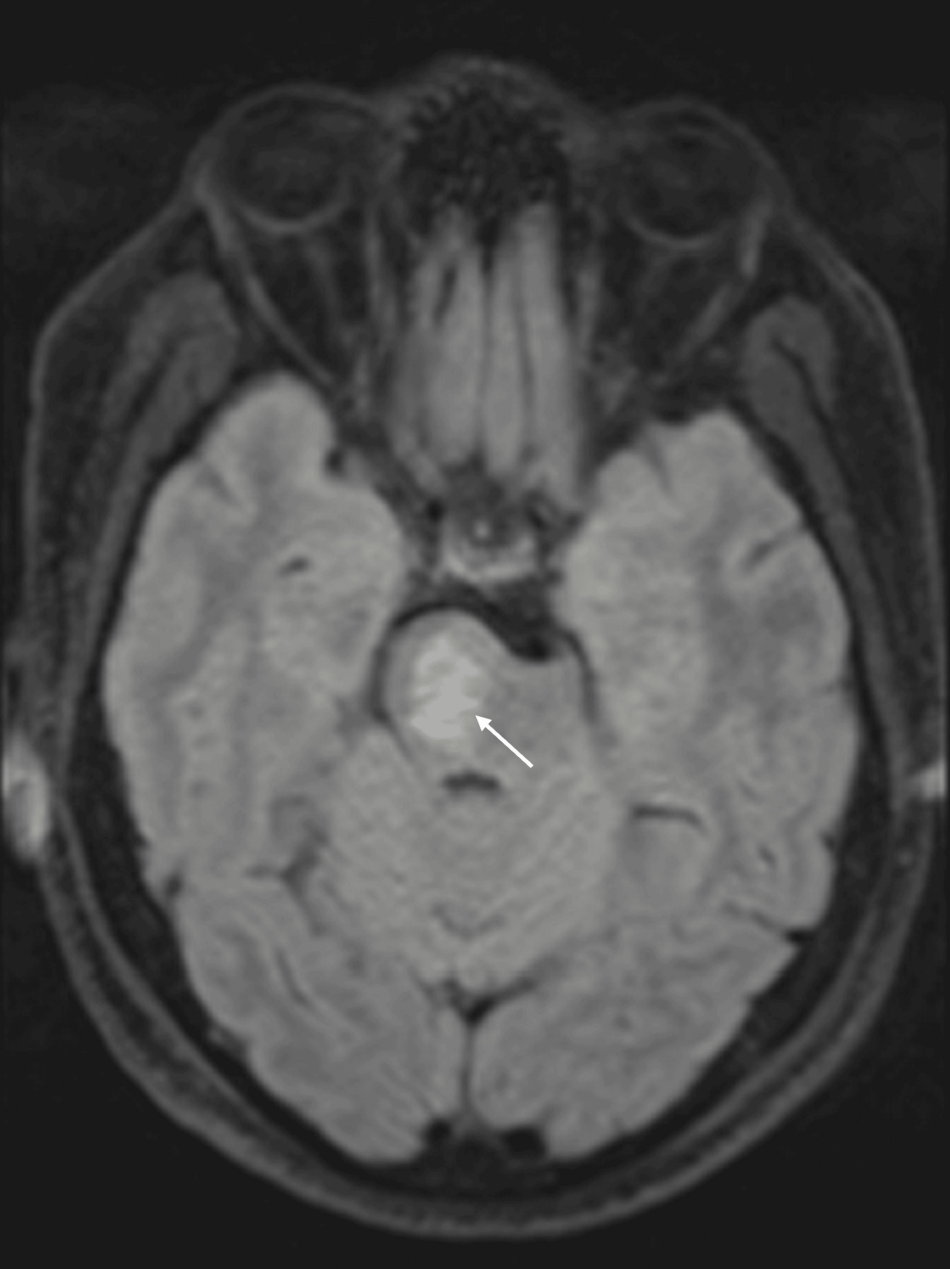
Brain MRI demonstrating an acute ischemic lesion in the right pons. Axial T2-FLAIR (fluid-attenuated inversion recovery) sequence showed a hyperintense lesion in the right aspect of the pons (arrow), compatible with an acute ischemic event and correlating with the patient’s left-sided motor deficits.

**Figure 4:**
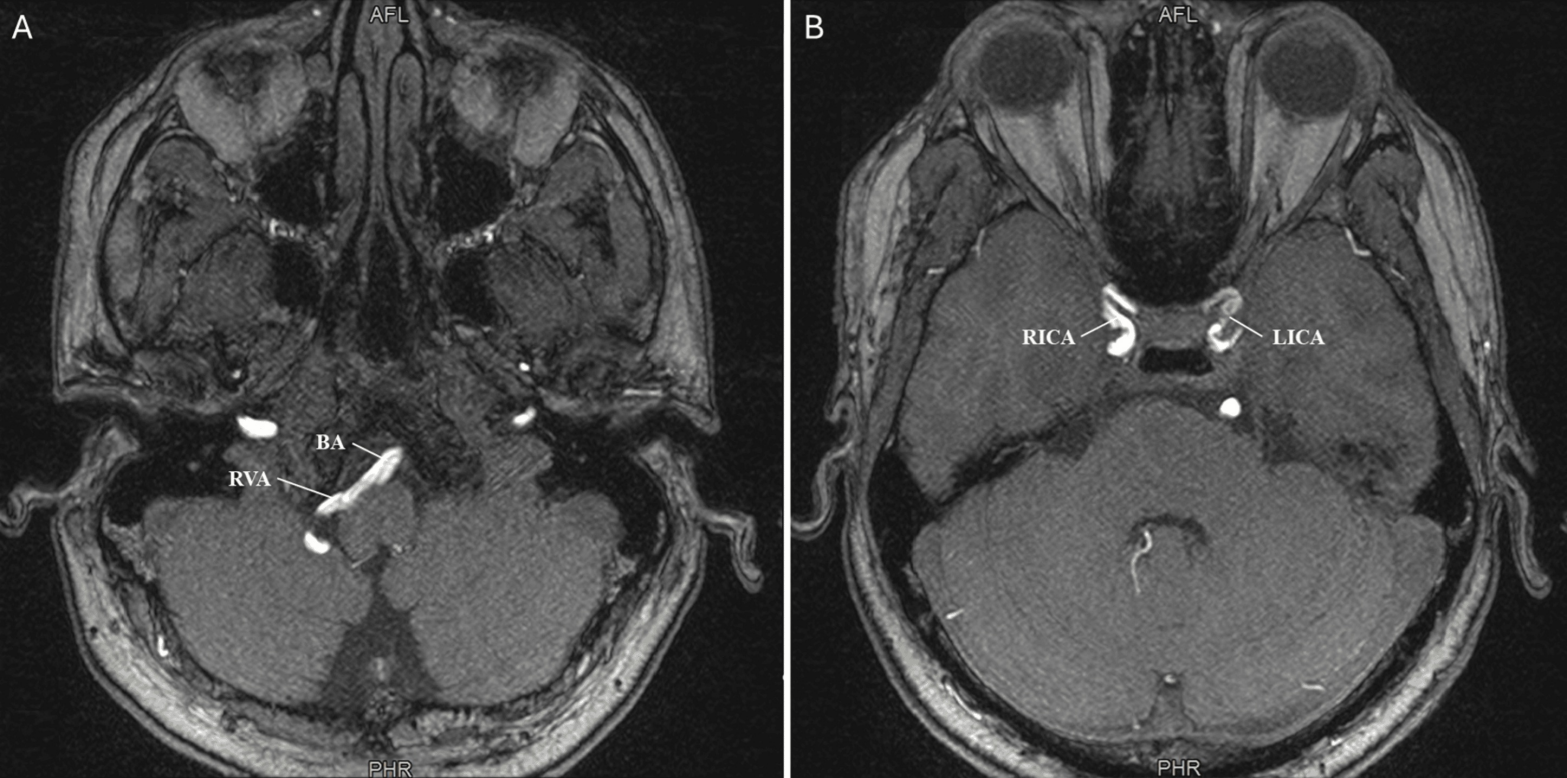
Magnetic resonance angiography showing absent flow in the left vertebral artery and reduced caliber of the left internal carotid artery. Magnetic resonance angiography demonstrated absent flow in the V4 segment of the left vertebral artery (panel A) and reduced caliber of the left internal carotid artery (panel B), findings consistent with multiterritorial large-vessel involvement. BA: basilar artery; LICA: left internal carotid artery; RICA: right internal carotid artery; RVA: right vertebral artery.

On the fifth day of admission, brain magnetic resonance imaging (MRI) revealed an ischemic lesion in the right aspect of the pons (Figure 3). Magnetic resonance angiography demonstrated absent flow in the V4 segment of the left vertebral artery and reduced caliber of the left internal carotid artery, findings suggestive of large-vessel involvement (Figure 4). Laboratory studies showed worsening renal function during the early hospital course after initiation of an angiotensin-converting enzyme inhibitor, with serum creatinine rising from 0.9 mg/dL on admission to 1.2 mg/dL, and estimated glomerular filtration rate decreasing from 111 to 79 mL/min/1.73 m², raising suspicion for renovascular disease. The thrombophilia panel, including protein C, protein S, antithrombin III, factor V Leiden, and prothrombin gene mutation, was negative. Acute-phase reactants were within normal limits (Table 1).

**Table 1:** Laboratory findings on admission. ESR: erythrocyte sedimentation rate; eGFR: estimated glomerular filtration rate; HbA1c: glycosylated hemoglobin; LDL-C: low-density lipoprotein cholesterol; HDL-C: high-density lipoprotein cholesterol; PT: prothrombin time; INR: international normalized ratio; aPTT: activated partial thromboplastin time; hs-CRP: high-sensitivity C-reactive protein; BNP: brain natriuretic peptide; TSH: thyroid-stimulating hormone.

Parameters	Patient values	Reference range
Hemoglobin	14.5 g/dL	13.0–17.5
Hematocrit	43%	40–52
White blood cells	7.2 ×10⁹/L	4.0–11.0
Neutrophils	60%	40–75%
Platelets	260 ×10⁹/L	150–400
ESR	8 mm/h	<20
Sodium	140 mmol/L	135–145
Potassium	4.1 mmol/L	3.5–5.1
Chloride	104 mmol/L	98–107
Urea	32 mg/dL	10–50
Creatinine	0.9 mg/dL	0.7–1.2
eGFR	>90 mL/min/1.73 m²	>60
Fasting glucose	92 mg/dL	70–99
HbA1c	5.2%	<5.7%
Total cholesterol	165 mg/dL	<200
LDL-C	92 mg/dL	<130
HDL-C	52 mg/dL	>40
Triglycerides	110 mg/dL	<150
PT	12.0 s	11–14
INR	1.0	—
aPTT	29 s	25–35
Fibrinogen	330 mg/dL	200–400
Protein C activity	105%	70–140%
Protein S activity	92%	60–140%
Antithrombin III	102%	80–120%
Factor V Leiden mutation	Negative	—
Prothrombin G20210A mutation	Negative	—
Antiphospholipid antibodies	Negative	—
Lupus anticoagulant	Not detected	—
Homocysteine	9 µmol/L	5–15
hs-CRP	0.8 mg/L	<3
Troponin I	<0.01 ng/mL	<0.04
BNP	28 pg/mL	<100
D-dimer	0.32 mg/L FEU	<0.5
Plasma renin activity	1.6 ng/mL/h	0.5–3.5
Aldosterone	9 ng/dL	4–31
Aldosterone/renin ratio	5.6	<20
TSH	1.4 µIU/mL	0.4–4.0
Free T4	1.1 ng/dL	0.9–1.7
Cortisol (AM)	14 µg/dL	5–25
Plasma metanephrines	Normal	Metanephrine <0.5; Normetanephrine <0.9 nmol/L
24-h urinary catecholamines	Normal	—
24-h urinary metanephrines	Normal	—
Urinalysis	Normal	—
Urine albumin/creatinine ratio	<10 mg/g	<30

**Figure 5:**
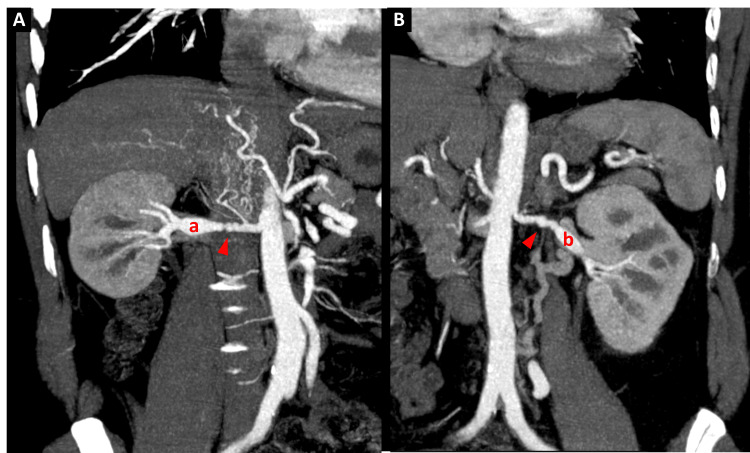
CT angiography of the renal arteries. Coronal reconstructions of the right renal artery (A) and left renal artery (B) demonstrated fibromuscular dysplasia with the characteristic “string-of-beads” appearance (arrowheads). a: right renal artery; b: left renal artery.

**Figure 6:**
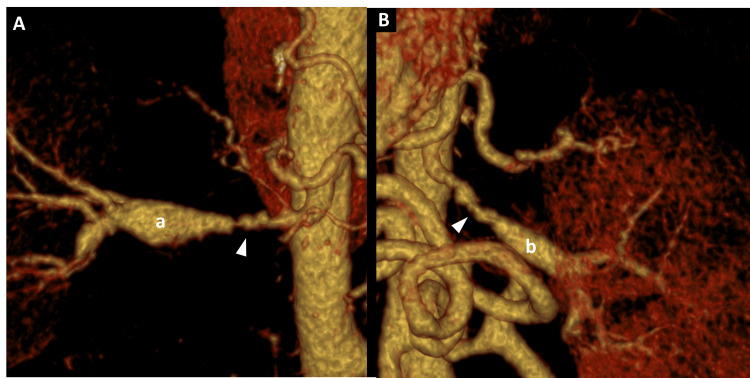
Three-dimensional reconstruction of CT angiography of the renal arteries. 3D coronal view of the right renal artery (A) and left renal artery (B) demonstrating fibromuscular dysplasia with the characteristic “string-of-beads” appearance (arrowheads). a: right renal artery; b: left renal artery.

Given the clinical picture of secondary hypertension and evidence of multiterritorial vascular involvement, CT angiography of the renal arteries was performed, revealing saccular dilatations along both arteries with the characteristic “string-of-beads” appearance, consistent with fibromuscular dysplasia (Figures 5, 6).

The patient was managed conservatively, with optimization of antihypertensive therapy using a combination of a calcium channel blocker and an angiotensin II receptor blocker. Antiplatelet therapy with aspirin and a statin was initiated. After multidisciplinary discussion with the nephrology team, endovascular intervention was deemed unnecessary, as renal function remained stable and blood pressure was adequately controlled with medical therapy (serum creatinine = 0.8 mg/dL, estimated glomerular filtration rate = 115 mL/min/1.73 m², and blood pressure = 134/75 mmHg at discharge). Motor rehabilitation was initiated, resulting in partial recovery of neurological deficits.

He was discharged with scheduled follow-up in neurology and internal medicine clinics, along with guidance for strict control of cardiovascular risk factors.

## Discussion

Although FMD can occur in both sexes, approximately 90% of cases are reported in females, typically between 15 and 50 years of age [4]. Consequently, the diagnosis of FMD in a young male patient, as in this case, is uncommon and warrants particular clinical attention.

The exact pathophysiology of FMD remains uncertain. Proposed mechanisms include genetic predisposition, hormonal influences, and mechanical stress on the arterial wall [3]. Histopathologically, FMD can be classified into three main types (medial, intimal, and adventitial), according to the predominant arterial layer affected. The medial type is the most common and is classically associated with the “string-of-beads” angiographic pattern [5], which reflects alternating segments of stenosis and aneurysmal dilatation resulting from fibroplasia and post-stenotic dilation.

The renal arteries are affected in approximately 60-75% of cases, frequently resulting in renovascular hypertension. Cerebrovascular involvement, particularly of the carotid and vertebral arteries, may manifest as migraine-like headache, pulsatile tinnitus, transient ischemic attacks, ischemic stroke, or subarachnoid hemorrhage [4,6,7]. The coexistence of renal and cerebrovascular FMD, as demonstrated in this patient, underscores the systemic nature of the disease. Indeed, the identification of lesions in one vascular territory should prompt imaging evaluation, from head to pelvis, with computed tomography angiography (CTA) or magnetic resonance angiography (MRA), as up to two-thirds of patients exhibit multivessel involvement [7-9].

Neurological manifestations of FMD, including ischemic stroke, typically result from arterial dissection, thromboembolism, or hemodynamic compromise secondary to stenosis [10]. Involvement of the posterior cerebral circulation, such as stenosis or occlusion of the vertebral artery, is less frequent than that of the anterior circulation but has been reported in several case series. Recognizing FMD as the underlying etiology is critical, as it may influence secondary prevention strategies and guide screening for extracranial vascular disease.

In this case, the presentation of a pontine ischemic stroke in a young man with long-standing, difficult-to-control hypertension prompted suspicion of a secondary cause. The subsequent rise in serum creatinine after initiation of an angiotensin-converting enzyme inhibitor further strengthened the suspicion of renovascular pathology. Secondary hypertension should always be considered in young patients with severe or refractory hypertension, hypertensive target-organ damage, or sudden worsening of previously stable blood pressure control [11].

Imaging studies play a fundamental role in establishing the diagnosis. CTA and MRA have largely replaced invasive methods for diagnostic purposes, providing excellent spatial resolution and enabling noninvasive vascular mapping [12]. However, in cases with strong clinical suspicion of FMD in which CTA or MRA do not reveal abnormalities, catheter-based angiography remains the gold standard for detecting arterial changes. This invasive study should only be performed if the results are expected to have a significant impact on patient management [13]. In this patient, CTA demonstrated bilateral renal artery involvement with the characteristic “string-of-beads” appearance, confirming multifocal FMD. Concurrent findings of vertebral artery occlusion and reduced internal carotid artery caliber supported systemic arterial involvement.

The therapeutic approach to FMD depends on clinical presentation, degree of stenosis, and associated complications. Medical therapy remains the cornerstone of management in asymptomatic patients or those with stable hemodynamics. Blood pressure control, particularly with agents targeting the renin-angiotensin system, is essential to limit the progression of target-organ damage [14]. Percutaneous transluminal angioplasty (PTA) is the treatment of choice for patients with resistant hypertension, progressive renal dysfunction, or intolerance to medical therapy, with success rates approaching 80% for improvement or normalization of blood pressure control [15]. However, given the stable renal function and satisfactory blood pressure control in this case, a conservative strategy was chosen after multidisciplinary discussion with the nephrology team.

The differential diagnosis of FMD primarily includes inflammatory arterial diseases, atherosclerosis, arterial vasospasm, and segmental arterial mediolysis (Table 2).

**Table 2:** Differential diagnosis of fibromuscular dysplasia. The table was created by the authors using original data synthesis and was not reproduced or adapted from any copyrighted source. FMD: fibromuscular dysplasia.

Condition	Key features
Segmental arterial mediolysis	Preferentially affects abdominal visceral arteries; may cause aneurysms, dissections, ruptures, or occlusions. Angiographic findings can be indistinguishable from FMD. Definitive diagnosis is histopathologic, characterized by vacuolar degeneration of the tunica media [16].
Arterial spasm	Benign and transient radiologic findings showing regular arterial vasospasm, distinct from the FMD “string-of-beads” pattern with beads of varying diameters. Spasm may be induced by ergotamine, sympathomimetics, or catheter manipulation [6,9].
Atherosclerosis	Associated with advanced age, alcoholism, smoking, diabetes mellitus, dyslipidemia, obesity, and hypertension. Imaging typically reveals calcified plaques. It mainly affects proximal arterial segments and bifurcation points.
Large-vessel vasculitides (giant cell arteritis; Takayasu arteritis)	Characterized by constitutional symptoms (fever, weight loss), elevated acute-phase reactants, anemia, and thrombocytopenia. Imaging demonstrates edematous thickening of the arterial wall, particularly in proximal segments [17,18].

The prognosis of FMD is generally favorable, although patients require long-term follow-up due to the risk of disease progression, development of new vascular lesions, aneurysm formation, or arterial dissection [14]. Imaging surveillance of the affected vascular territories is recommended, along with clinical monitoring of blood pressure and renal function. Lifestyle modification and optimization of cardiovascular risk factors further contribute to a favorable outcome.

## Conclusions

FMD should be considered in the differential diagnosis of secondary hypertension, particularly in young or middle-aged adults who present with difficult-to-control hypertension or evidence of target-organ damage in the absence of atherosclerotic risk factors. This case illustrates the diagnostic challenge posed by FMD, which may involve multiple vascular territories and present with diverse clinical manifestations, including cerebrovascular ischemic events. A systematic approach, integrating clinical suspicion, targeted laboratory evaluation, and advanced vascular imaging, is essential to ensure timely recognition of the disease.

Therapeutic strategies must be individualized, balancing the risks and benefits of revascularization against the effectiveness of medical therapy. In patients with stable renal function and satisfactory blood pressure control, conservative pharmacologic treatment remains an appropriate option. This case reinforces the importance of maintaining vigilance for secondary causes of hypertension and highlights the value of multidisciplinary collaboration between internal medicine, nephrology, and neurology to optimize clinical outcomes. Early diagnosis and appropriate follow-up are critical to preventing complications and improving prognosis in FMD.
